# Arachnoid Cysts in Athletes with Sports-Related Concussion: A Case Series and Literature Review

**DOI:** 10.1186/s40798-024-00757-x

**Published:** 2024-09-02

**Authors:** Andrew R. Stevens, Kamal M. Yakoub, David J. Davies, Antonio Belli, Philip J. O’Halloran

**Affiliations:** 1 Neuroscience and Ophthalmology, Institute of Inflammation and Ageing, Robert Aitken Institute for Clinical Research, University of Birmingham, Edgbaston, Birmingham, B15 2TT UK; 2 Department of Neurosurgery, Queen Elizabeth Hospital Birmingham, Mindelsohn Way, Edgbaston, Birmingham, B15 2GW UK; 3 Royal College of Surgeons of Ireland, Dublin, Ireland

**Keywords:** Arachnoid cyst, Sports-related concussion, Concussion, Mild traumatic brain injury, Traumatic brain injury, Cyst rupture, Athletes, Sports injury, Subdural haematoma, Post-concussion syndrome

## Abstract

**Background:**

Arachnoid cysts (AC) are associated with a risk of rupture or haemorrhage following head impact and pose a potential predisposing factor for significant complications of sport-related concussion. Despite a recognised association between ACs and intracranial haemorrhage/cyst rupture, the risk profile of participating in contact sports with AC is not well defined. We report a retrospective case series of players presenting to the Birmingham Sports Concussion Clinic between 2017 and 2023 and underwent MRI head, with a comprehensive review of the prior literature.

**Results:**

432 athletes underwent MRI of which 11 were identified to have AC (middle fossa n = 8; posterior fossa n = 2, intraventricular n = 1). Average maximal diameter was 4.1 ± 1.2 cm. 64% had a protracted recovery (≥ 3 months). 9% experienced an AC specific complication (cyst rupture, complete neurological recovery, maximal diameter 6.5 cm, Galassi II, 4 previous concussions). 91% of patients (mean maximal diameter 3.9 ± 1.0 cm) experienced no complications despite multiple previous accumulated sports-related concussions (mean 3.3, range 1–9). Case studies from the literature are summarised (n = 63), with 98% reporting complications, none of which resulted in adverse or unfavourable neurological outcomes. Across prospective and retrospective cohort studies, 1.5% had a structural injury, and (where outcome was reported) all had a favourable outcome.

**Conclusions:**

AC is an incidental finding in athletes, with the majority in our cohort having sustained serial concussions without AC complication. The single complication within this cohort occurred in the largest AC, and AC size is proposed as a tentative factor associated with increased risk of contact sports participation. Complications of AC appear to be a rare occurrence. This case series and review has not identified evidence to suggest that participation in sports with AC is of significant risk, though individualised assessment and discussion of the potential risks of contact sports participation should be offered.

## Key Points

- Incidental arachnoid cysts were found to occur in 2.5% of athletes presenting after sports-related concussion.
- Of 11 athletes with AC and a history of concussion, one (9%) had experienced a prior complication of AC (cyst rupture) and made a full recovery.
- A comprehensive review of the prior literature suggests an overall risk of complications of 1.5%, with all reported outcomes from cases describing a full neurological recovery.
- Overall, complications of AC in athletes after sport-related concussion appear to be rare, with no recorded cases of significant neurological consequences.

## Background

After incidental discovery of an arachnoid cyst (AC) in athletes, appropriate risk management and clinical advice on continued participation in contact sports is an area of mixed opinions [1, 2]. Whilst ACs are typically asymptomatic congenital lesions; haemorrhage or cyst rupture secondary to modest mechanical forces on the brain are possible outcomes, potentially requiring surgical intervention and may result in long-term neurological deficits [3, 4].

Neuroimaging is increasingly common amongst athletes who play contact sports, and consequently the identification of incidental AC is a commonly encountered scenario. Despite a recognised association between ACs and intracranial haemorrhage/cyst rupture, the risk profile of participating in contact sports with AC is not well defined, and there is no consensus opinion on the appropriate management [5–7]. The medical literature describes many cases of structural brain injury associated with AC, yet few case series or observational studies describe the natural history of athletes who have participated in, or continue to participate in, contact sports. We present a case series of all players with AC attending our sports concussion clinic over a 6 year period, followed by comprehensive summary of the literature to date.

## Methods

This retrospective case series included athletes presenting to the Birmingham Concussion Clinic between 2017 and 2023. Of all athletes requiring MRI head, reports were screened to identify all with AC.

This cohort of players encompasses athletes which were deemed by their primary health provider to require specialist evaluation. Referral pathways vary, and may be directly from medical teams of sports clubs or via a general practitioner. There are no strict guidelines on referral, though athletes are typically referred in three scenarios, as a result of: serial concussion; prolonged post-concussion syndrome; or other concerning clinical presentation after recent or historical concussion(s). Athletes are able to access the clinic from any level of any sport (amateur to professional) and encompass players from adolescence onwards (age 14+).

Patients were retrospectively identified based on coding records of clinic attendance and search of MRI reports. No specific guidelines to indicate need for MRI, but the majority of imaging are performed on the basis of: symptoms persisting over four weeks post-injury (including subjective cognitive disturbance); significant (dependent on age/sport/competition level) concussion history; concerning signs immediately post-injury (e.g. ataxia); or concerns of a low threshold for symptomatic concussion based on descriptions and/or video footage of impact.

MRI was performed using a 3T Magnetom Skyra (Siemens, Munich, Germany), with acquisition of T1, T2, T2*, FLAIR, 1H-MR spectroscopy, resting state functional MRI, diffusion tensor imaging and MR elastography. AC dimensions were measured using integrated functions in Caresteam (Carestream Health, New York, USA) and recorded by two competent practitioners.

After identification, data were extracted retrospectively from electronic patient records and imaging, including: patient demographics; number of previous concussions; history, classification and dimensions of AC; and clinical outcomes. History of previous concussion were taken retrospectively based on patient reporting, validated by contemporaneous records kept by the athlete’s medical team, where available.

A review of the existing literature was performed on 24th November 2023, using the Medline, EMBASE, Web of Science and Google Scholar, using terms related to “traumatic brain injury” or “concussion” appearing with “arachnoid cyst” or “cyst”. The date range for each database was from inception to the date of search. These searches were supplemented by a review of the reference lists of related works, including previous reviews seeking to comprehensively report case studies in this area [8].

Inclusion criteria were: (1) studies of all designs; (2) studies which reported cases or cohorts of athletes with AC. Exclusion criteria were: (1) cases or cohorts where there is no record of or reference to sport participation. Studies were excluded which reported spontaneous complications, or as a result of concussion or head impact related to non-sporting activities (such as road traffic collisions, assaults, non-sporting falls or impacts relating to other non-sporting recreational activities). Notably, the search was not limited to cases reporting complications: studies reporting the natural history of athletes with AC were included.

After study identification and removal of duplicates, clinical data were extracted and presented in tabular form and as a narrative review. Specifically, the following information was extracted from the selected studies: author(s); year of publication; gender; age; sports participation history; injury mechanism; AC anatomical location and dimension; and detail of any complications, including clinical presentation; management; outcome and return to play (RTP).

## Results

### Case Series

432 athletes underwent MRI (576 MRIs performed in total) of whom 11 were identified to have AC (temporal (Galassi I) n = 1, temporal (Galassi II) n = 7; posterior fossa n = 2, intraventricular n = 1) (Table 1). Of players with identified AC, the mean age was 22 years, with a male:female ratio of 10:1.

**Table 1:** Summary of 11 cases of AC presenting to a sports concussion clinic

Case	Age	M/F	Sport	Previous concussions	AC location	Galassi	Scalloping	Dimensions (cm)	Complications
1	16–24	M	Rugby	2	MF	2	No	4.5 × 1.8 × 3.0	Nil
2	16–24	M	Rugby	9	MF	2	No	4.2 × 1.9 × 3.2	Nil
3	16–24	F	Soccer	1	MF	2	No	4 × 3.2 × 4.5	Nil
4	25–30	M	Rugby	6	PF	N/A	Yes	4.2 × 5.8 × 5	Nil
5	16–24	M	Rugby	2	MF	2	No	3.8 × 2.5 × 3.3	Nil
6	16–24	M	Rugby	1	IV	N/A	N/A	2.8 × 3 × 2.1	Nil
7	25–30	M	Cricket	2	PF	N/A	Yes	1.7 × 2.2 × 3.4	Nil
8	31–35	M	Rugby	3	MF	2	No	4 × 3.4 × 3	Nil
9	16–24	M	Rugby	4	MF	1	No	1.5 × 2.1 × 0.5	Nil
10	16–24	M	Soccer	4	MF	2	No	6.5 × 4.5 × 3.8	Previous rupture
11	16–24	M	Martial Arts	3	MF	2	Yes	2.7 × 3.7 × 3.1	Nil

MF, middle fossa; PF, posterior fossa; IV, intraventricular; dimensions given as antero-posterior × lateral × supero-inferior; age given at diagnosis of AC. Age range given in lieu of specific age as a measure to ensure anonymity

Reasons for presentation to the clinic varied. 7/11 presented with persistent symptoms, either at rest (5/11) or on exercise (2/11). 6/11 presented with headache as the predominant symptom, 1/11 with blurred vision and fatigue. Of n = 7 presenting with symptoms, median time to symptom resolution was 3 months (IQR 2–4.5 months). 2/11 presented after incidental discovery of the AC (without recent concussion following imaging for other indications), though both had previously sustained at least one concussion during their career. 2/11 presented without persistent symptoms, but were referred for assessment following repeated concussions in a short interval.

All 11 players had a single AC only. Average maximal diameter of AC was 4.1 ± 1.2 cm. 7/11 had a protracted recovery (time to symptom resolution (SR) or return to play (RTP) ≥ 3 months). One athlete had experienced previous AC specific complications (maximal diameter 6.5 cm, Galassi II, 4 previous concussions): the patient had undergone previous cyst fenestration due to pressure-related headache several years prior to presentation at our clinic. After the primary surgical intervention, the player had made a complete recovery and had returned to sport. The player sustained one further concussion and presented to our clinic with headaches, which were not associated with further rupture or structural brain injury when evaluated.

**Fig. 1:**
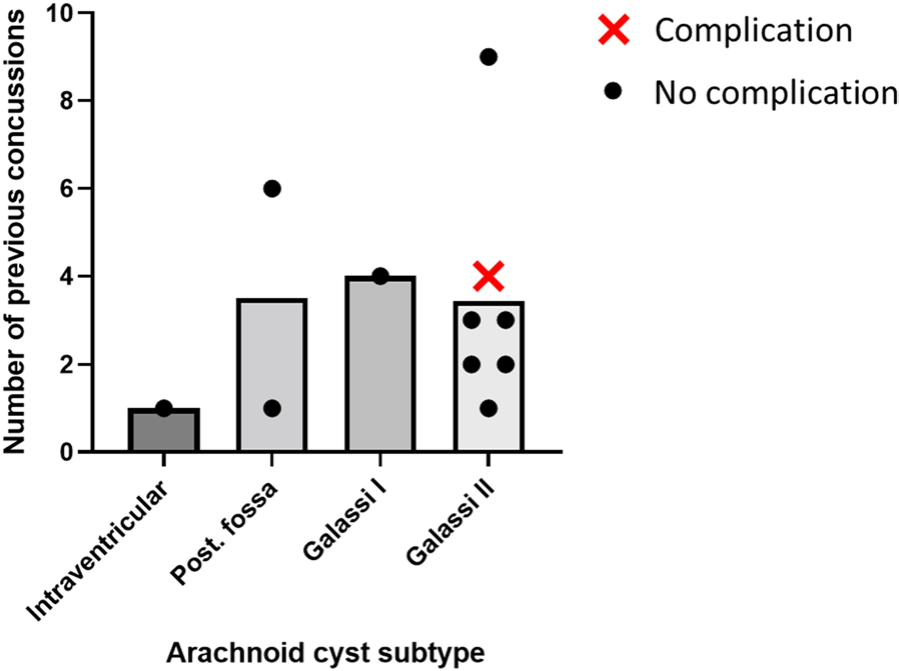
Cases represented by number of previous concussions (mean with individual cases represented as dots/crosses for no complication/complication respectively) and AC subtype

10/11 patients (mean AC maximal diameter 3.9 ± 1.1 cm) had experienced no complications despite sustaining previous accumulated sports-related concussions (mean 3.3 ± 2.4). 5/11 continued to play their sport after discovery of the AC. Sports represented were rugby union (7/11); football (soccer) (2/11); cricket (1/11); and martial arts (1/11). Players competed across all levels: 3/11 professional; 3/11 semi-professional; 5/11 amateur. Amateur levels also encompassed local club level to competing at a national amateur level. In total, players without a history of complications of AC had experienced 33 concussions over their combined playing careers. Distribution of previous concussion history according to AC type is shown in Fig. 1. Players ages ranged from 17 to 33, and whilst not all career histories were available, most had commenced their sporting careers as children.

6/11 returned to play (RTP) (median RTP time 3 months (IQR 3–9 months). 2/11 had prolonged RTP due to repeated concussions and a recommendation of an extended break, irrespective of AC diagnosis. 3/11 had prolonged RTP due to persistent symptoms and failure to progress with graduated exercise. 1/11 had prolonged RTP due to a combination of factors including AC diagnosis and other personal circumstances. Following individualised discussions, 5/11 players opted to retire from competitive contact sports.

### Literature Review

Case series and reports were identified which reported athletes whom sustained a concussion which resulted in structural brain injury associated with AC. Table 2 summarises the details from these 62 cases. 11/62 cases resulted in subdural hygroma associated with cyst rupture; 51/62 cases presented with subdural haematoma (all chronic or subacute). 5/62 cases were managed conservatively, with remaining cases undergoing burr hole evacuation or craniotomy. No case was reported which resulted in adverse or unfavourable neurological outcomes. 3/62 cases required subduro-peritoneal shunt. A broad spectrum of sports were represented amongst the cases: football (soccer) 23/62; cycling 6/62; martial arts 4/62; football (American) 3/62; winter sports 3/62; basketball 3/62; and boxing 2/62. 3/62 were not specified and the remainder (14/62) were from a variety of sports as described in Table 2.

**Table 2:** Summary data from all identified structural brain injuries reported in the literature in case-reports or case-series where the complication is deemed related to sports participation

	References	Year	Age	M/F	Sport	AC	Type/size	Mechanism of injury details	Symptoms	Time post-injury	Structural injury	Management	Outcome	RTP
1	Oliver [10]	1958	21	M	Football (soccer)	MF	NR	Severe blow to the chest playing soccer	Headache and vomiting	10 weeks	Intracystic haemorrhage	Craniotomy and excision	Complete recovery	NR
2	Weinberg and Flom [11]	1973	20	M	Judo	MF	Galassi III*	Thrown several times	Headache	2 days	SDH/intracystic haemorrhage	Craniotomy and drainage	NR	NR
3	Lacour et al. [12]	1978	13	F	Water-skiing	MF	Galassi II*	Several falls whilst water-skiing	Headaches, vomiting, leg weakness	4 weeks	SDH	Craniotomy, evacuation and fenestration	NR	NR
4	Varma et al. [13]	1981	17	M	Rugby	MF	NR	Head impact during tackle	Headache	7 weeks	SDH	Craniotomy, evacuation and fenestration	Uneventful recovery	NR
5	Cullis and Gilroy [14]	1983	11	M	Swimming^@^	MF	Galassi III*	No specific event	Headaches	4 weeks	SDH	Craniotomy and drainage	NR	NR
6	Hara et al. [15]	1984	13	M	Cycling	MF	Galassi III*	Fall from 2 m	Headache and nausea	10 weeks	SDH/intracystic haemorrhage	Craniotomy, evacuation and fenestration	Complete recovery	NR
7	McNeil et al. [16]	1987	17	M	Break dancing	MF	Galassi I*	Head impacts whilst spinning on the head	Headache	12 weeks	SDH/intracystic haemorrhage	Burr hole	Complete recovery	Retired
8	Page et al. [17]	1987	57	M	Horse riding	MF	Galassi II*	Thrown from horse	Headache and nausea	7 days	Contralateral SDH	Craniotomy and fenestration	Ongoing headache	NR
9	Yokoyama et al. [18]	1988	17	M	Judo	MF	Galassi II*	Fell down and hit head	Headache and nausea	8 weeks	SDH/intracystic haemorrhage	Craniotomy, evacuation and fenestration	NR	NR
10	Kulali and von Wild [19]	1989	6	M	Cycling	MF	Galassi II*	Bicycle accident	Headache and vomiting	4 weeks	Subdural hygroma	Craniotomy and drainage	Ongoing headache and required subduro-peritoneal shunt	NR
11	Kulali and von Wild [19]	1989	15	M	Dancing	MF	Galassi I*	Sudden onset whilst dancing	Headache and vomiting	3 weeks	Subdural hygroma	Craniotomy and drainage	Uneventful recovery	NR
12	Maeda et al. [20]	1993	14	M	Football (soccer)	MF	Galassi II*	Headache after playing soccer	Headache	3 days	SDH/intracystic haemorrhage	Craniotomy, evacuation and fenestration	NR	NR
13	Ochi et al. [21]	1995	12	M	Physical training	IH	N/A	Minor trauma	NR	NR	SDH/intracystic haemorrhage	NR	NR	NR
14	Albuquerque and Gianotta [22]	1997	6	M	Rollerblading	MF	NR	Fall	Headache, lethargy and nausea	NR	Subdural hygroma	Craniotomy and drainage, subseqeunt subduro-peritoneal shunt	Good recovery	NR
15	Vigil et al. [23]	1998	16	M	Football (American)	MF	Galassi II*	Several hard hits during game with post-game headache, played several further games with recurrent headache	Headache	2 weeks	Subdural hygroma	Burr hole drainage, subsequent repeat drainage, then fenestration	Good recovery	Retired
16	Kawanishi et al. [24]	1999	14	M	Football (soccer)	MF	NR	Heading the ball	Headache	7 weeks	SDH	Burr hole	Reduction in cyst size after 1 year	NR
17	Kawanishi et al. [24]	1999	11	M	Football (soccer)	MF	Galassi II*	Heading the ball	Headache and vomiting	2 days	SDH	Burr hole	Symptoms resolved	NR
18	Donaldson et al. [25]	2000	14	M	Football (American)	MF	Galassi II*	Collision playing football	Headache	4 weeks	Subdural hygroma	Craniotomy, drainage and fenestration	Good recovery	Yes, asymptomatic playing for two years after
19	Chillala et al. [26]	2001	21	M	Football (soccer)	MF	Galassi III*	Headed the ball several times in a row	Headache	1 day	SDH	Drainage (not specified)	Uneventful recovery	NR
20	Prabhu et al. [27]	2002	16	F	Football (soccer)	MF	3 cm diameter	Struck by soccer ball with significant force	Headache and paraesthesia	1 month	SDH	Craniotomy, evacuation and fenestration	Uneventful recovery	NR
21	Prabhu and Bailes [27]	2002	16	F	Football (soccer)	MF	Galassi II*	Struck by soccer ball with significant force	Headache and paraesthesia	4 weeks	SDH	Craniotomy, evacuation and fenestration	Complete recovery	NR
22	Mori et al. [28]	2002	14	M	Physical training	MF	Galassi I* (1.5 × 0.7 cm)	Fall	Headache and hemiparesis	4 weeks	SDH/intracystic haemorrhage	Burr hole	Symptoms resolved	NR
23	Gelabert Gonzalez [29]	2002	13	M	Football (soccer)	MF	Galassi II*	Mild head injury	Headache	15 days	Subdural hygroma	Craniotomy, drainage and fenestration	Uneventful recovery	NR
24	Ulmer et al. [30]	2002	44	M	NR (During sports)	MF	Galassi III ("giant")	Fell onto head	Headache, nausea, cognitive impairment	Months	Subdural hygroma	Craniotomy, drainage and fenestration, shunt and repeated needle aspirations	Good recovery	NR
25	Tsuzuki et al. [31]	2003	16	F	Basketball ^@^	MF	Galassi II*	No specific event	Headache	"Several days"	SDH	Burr hole	Uneventful recovery	NR
26	Demetriades et al. [32]	2004	24	NS	Football (soccer)	MF	Galassi II*	Repeated head trauma after heading the ball	Headache and nausea, unsteadiness and right sided weakness	6 weeks	SDH	Burr hole	Satisfactory recovery	NR
27	Pretorius and McAuley [33]	2005	11	M	Outdoor pursuits^@^	MF	Galassi III*	No specific event	Headache	Several weeks	SDH/intracystic haemorrhage	Craniotomy and evacuation	NR	NR
28	Robles & Hernandez [34]	2006	20	M	Amateur boxing	MF	Galassi III*	Dizziness during boxing bout	Headache and dizziness, vomiting	1 month	SDH/intracystic haemorrhage	Craniotomy, evacuation and fenestration	Uneventful recovery	NR
29	Offiah et al. [35]	2006	8	M	Football (soccer)	MF	Galassi II*	Hit head on floor	Headache, vomiting, diplopia	Several weeks	Subdural hygroma	Burr hole drainage	Required subduro-peritoneal shunt then recovered well	NR
30	Bristol et al. [36]	2007	17	M	Football (American)	MF	Galassi II*	Helmet to helmet collision	Headache, eye pain, tunnel vision	3 days	Subdural hygroma	Craniotomy, drainage and fenestration	NR	NR
31	Tsitsopoulos et al. [37]	2008	15	M	Athletics	MF	Galassi III*	Head injury	Headache	10 days	SDH/intracystic haemorrhage	Craniotomy, evacuation and fenestration	Uneventful recovery	NR
32	Pillai et al. [38]	2009	23	M	Cycling	MF	Galassi II	Fall from bicycle	Headache and nausea	NR	SDH	Craniotomy, evacuation and fenestration	Complete recovery	NR
33	Domenicucci et al. [39]	2009	7	M	Football (soccer)	MF	NR	NR	NR	NR	SDH	CSDH drainage alone	Good recovery	NR
34	Domenicucci et al. [39]	2009	41	M	Cycling	MF	NR	NR	NR	NR	SDH	CSDH drainage alone	Good recovery	NR
35	Hamada et al. [40]	2010	15	M	Volleyball	MF	NR	Blow to the head by a volleyball	Headache and vomiting	Several weeks	SDH	Craniotomy, evacuation and fenestration	Uneventful recovery	NR
36	Zeng and Lin [41]	2011	14	M	Athletics	MF	Galassi III*	Head injury during "jump training"	Headache, vomiting, drowsiness	4 weeks	SDH/intracystic haemorrhage	Craniotomy, evacuation and fenestration	Complete recovery	NR
37	Zeng and Lin [41]	2011	16	M	Football (soccer)	MF	Galassi II*	Heading the ball during football training	Headache and nausea	4 weeks	SDH	Burr hole	Complete recovery	NR
38	Kertmen et al. [42]	2012	12	M	Taekwondo	MF	Galassi II*	Repetitive mild injury during training	Headache	2 weeks	SDH	Burr hole	Uneventful recovery	NR
39	Seddighi et al. [43]	2012	23	M	Cycling	MF	Galassi III* ("giant")	Fall from bicycle	Headache and vomiting	3 days	EDH	Craniotomy and evacuation	NR	NR
40	Zheng et al. [44]	2013	19	M	Sport related	MF	NR	Sport related	Headache	NR	SDH	Burr hole	No recurrence	NR
41	Zheng et al. [44]	2013	16	M	Sport related	MF	NR	Sport related	Headache and dizziness	NR	SDH	Burr hole	No recurrence	NR
42	Maher et al. [45]	2013	12	F	Football (soccer)	MF	Galassi II*	Minor head injury	Headache and vomiting	Several days	Subdural hygroma	Conservative management	Resolved	NR
43	Maher et al. [45]	2013	16	M	Football (soccer)	MF	Galassi II*	Minor head injury	Headache	2 weeks	Subdural hygroma	Conservative management	Reduced in size	NR
44	Hou et al. [46]	2014	17	M	Basketball ^@^	MF	Galassi II*	No specific event	Headache and dizziness	5 days	SDH	Burr hole	Symptoms resolved	NR
45	Edmondson et al. [47]	2014	14	M	Football (soccer)	MF	NR	Fall during practice	Headache and vomiting	10 weeks	SDH	Burr hole	Good	NR
46	Pascoe et al. [48]	2015	43	M	Football (Australian rules^#^)	MF	Galassi III*	Collision playing football	Headache and diplopia	2 weeks	SDH/intracystic haemorrhage	Craniotomy, evacuation and fenestration	Uneventful recovery	NR
47	Takizawa et al. [49]	2015	13	M	Cycling	MF	NR	Sport related	Headache	7 weeks	SDH	Burr hole	NR	NR
48	Takizawa et al. [49]	2015	15	M	Football (soccer)	MF	NR	Sport related	Headache	8 weeks	SDH	Craniotomy	NR	NR
49	Takizawa et al. [49]	2015	31	M	Judo	Mf	NR	Sport related	Headache	4 weeks	SDH	Burr hole	NR	NR
50	Takizawa et al. [49]	2015	32	M	Snowboarding	MF	NR	Sport related	Headache	20 weeks	SDH	Craniotomy	NR	NR
51	Takizawa et al. [49]	2015	35	F	Skiiing	MF	NR	Sport related	Headache, low GCS [8]	16 weeks	SDH	Burr hole	NR	NR
52	Rashid et al. [50]	2016	7	M	Trampolining	MF	Galassi II*	Fall from trampoline	Headache, agitation, paraesthesia	2 weeks	SDH/intracystic haemorrhage	Evacuation (not specified)	Dramatic recovery	NR
53	Yaldiz et al. [51]	2016	15	M	Football (soccer)	MF	Galassi III*	Minor head trauma	Headache	2 weeks	SDH	Burr hole	No complaints	NR
54	Wu et al. [52]	2017	17	M	Basketball	MF	NR	NR	NR	NR	SDH/intracystic haemorrhage	Burr hole	Favourable	NR
55	Furtado et al. [53]	2019	8	M	Football (soccer)	MF	Galassi II	Concussion during football match	Headache	30 days	SDH	Craniotomy, evacuation and fenestration	No adverse events	NR
56	Gregori et al. [54]	2020	10	M	Football (soccer)	MF	Galassi I*	Concussive head trauma	Headache	20 days	SDH	Burr hole	Good	NR
57	Gregori et al. [54]	2020	18	M	Football (soccer)	MF	Galassi III*	Concussive head trauma	Nausea and headache	10 days	SDH	Burr hole	Good	NR
58	Gregori et al. [54]	2020	6	M	Football (soccer)	MF	Galassi I*	Minor head trauma after heading the ball	Headache and nausea	15 days	SDH	Craniotomy	Good	NR
59	Beretta et al. [55]	2020	In 20 s	M	Football (soccer)	MF	Galassi I	Playing soccer	Headache	3 weeks	SDH	Burr hole	Complete recovery	NR
60	Benek and Ackay [56]	2021	26	M	Skiing	MF	NR	NR	NR	NR	SDH	Conservative management	Good recovery	NR
61	Benek and Ackay [56]	2021	21	M	Football (soccer)	MF	NR	NR	NR	NR	SDH	Conservative management	Good recovery	NR
62	Borni et al. [57]	2023	16	M	Amateur boxing/martial arts	MF	Galassi I (3.7 × 2.6 cm)	Repeated head impact	Headache	2 months	SDH	Conservative management	Lost to follow up	Advised to retire

CSDH, chronic subdural haematoma; MF, middle fossa; IH, interhemispheric; N/A, not applicable; NR, not recorded; SDH, subdural haematoma; RTP, return to play

*Extrapolated from images available in the publication

^#^Sport subtype inferred from context of country of origin of publication

^@^Sports participation without specific head impact recorded. Time post-injury refers to the time period between index injury and identification of any reported complication

Table 3 presents summary details of the single reported case of a player with a significant previous history of engagement in elite contact sports (American football) with a large AC, with no identified complication or associated structural brain injury.

**Table 3:** Summary data from all cases with identified AC reported in the literature in case-reports or case-series where sports participation has not resulted in structural brain injury

Reference	Year	Age	M/F	Sports participation	Type	Arachnoid cyst size	Previous head injury history	Adverse events	Management
Gamradt et al. [58]	2008	32	M	Professional Football (American)	MF	7.5 × 5.5 × 4.1 cm (Galassi II*)	More than 20 years of competitive American football experience. At no time in his career had he experienced a concussion or intracranial symptoms	Nil	Continue play

MF, middle fossa; IH, interhemispheric; NR, not recorded; SDH, subdural haematoma; N/A, not applicable

*Extrapolated from images

Prospective studies available in the literature are limited to paediatric cohorts (Table 4) [1, 9]. During their follow up periods, the combined prevalence of structural brain injury in the AC cohorts was 5/301 (1.7%). Both prospective cohorts included paediatric sports players with AC which had required previous surgical treatment, though the prevalence of rupture/haemorrhage within this subgroup were indeterminable from the publication. Three retrospective observational studies were identified, also all including paediatric only cohorts (Table 5). The combined prevalence of structural brain injury across these three studies was 3/244 (1.2%). Across prospective and retrospective cohort studies, 8/545 (1.5%) had a structural injury, and (where outcome was reported) all had a favourable outcome. As a scoping review, no formal risk of bias assessment was performed. No concerns were identified in the quality of the identified observational studies.

**Table 4:** Summary of prospective observational studies reporting incidence of structural brain injury in athletes with AC

References	Year	n =	Cohort type	Follow up period	Sports participation	Incidence of concussion	Adverse events	Outcomes	Notes
Strahle et al. [1]	2016	112	Paediatric	15.9 ± 8.8 months	All sport	18	2 (neurological symptoms with hygroma)	Full recovery in both	Approximately 15% were surgically treated before
Lee et al. [9]	2023	189	Paediatric	2700.5 seasons total (average 3.57 years per player)	All sport	44	3 sports related rupture or haemorrhage	No requirement for surgery, no lasting deficits	200 seasons (600 months of play) were after surgery

MF, middle fossa

**Table 5:** Summary of retrospective observational studies reporting incidence of structural brain injury in athletes with AC

References	Year	n =	Cohort type	Sports participation	Adverse events	Management	Outcome
Cress et al. [59]	2013	232	Paediatric	Mixed mild head trauma and sport	1 sport related rupture	NR	NR
Ellis et al. [60]	2015	3	Paediatric	Ice hockey (1); baseball (1); not specified (1)	1 occipital intracerebral haemorrhage (with MF AC)	Conservatively managed	Symptomatic of SRC for 144 days
Rogers et al. [61]	2016	9	Paediatric	Sport (not specified)	1 (cerebral contusion and subarachnoid haemorrhage)	Conservatively managed	Not reported

MF, middle fossa; NR, not recorded; SDH, subdural haematoma; SRC, sports-related concussion

## Discussion

We present a cohort of 11 athletes (from a range of sports and competition levels) with AC identified through attending our sports concussion clinic, amongst which 1 had experienced previous rupture requiring surgical intervention and made a good recovery. Amongst those without complication, 10 players had experienced a cumulative 33 documented concussions with no apparent previous incidence of clinical symptoms or radiological findings to indicate any adverse sequelae associated with AC. This case series is, to our knowledge, the largest of its kind for adult athletes. Our case series is supplemented by an extensive review of the existing literature on AC in athletes, including case reports, case series and observational studies. Though the majority of evidence is limited to paediatric cohorts, overall incidence of complications in athletes with AC was low (1.5%). Whilst outcomes were not reported in all cases, no case report or recorded outcome from observational or case studies has indicated that any athlete had persistent neurological deficits after an AC complication.

Arachnoid cysts are typically congenital collections of cerebrospinal fluid within an arachnoid membrane, with a prevalence of around 1.4% in the general population and are more common in males [62, 63]. In the majority of cases, ACs have a benign natural history with no adverse consequences. Brain development around the cyst is functionally normal, and no surgical intervention or treatment is required [62, 63]. As described in a number of case reports [2], rupture into the subdural space or associated haemorrhage after head impact is possible, resulting in a structural brain injury which may require surgical intervention. This may include evacuation of haemorrhage, decompression of a hygroma or cyst cavity, fenestration of the cyst into the subarachnoid space or, occasionally, insertion of a subdural-peritoneal shunt [22, 30, 35].

Increased recognition of adverse sequelae from concussion from participation in contact sports has resulted in an increase in neuroimaging in athletes, and consequently, increased diagnosis of AC as an incidental finding in this population. Concussion is a common phenomenon associated with considerable short and long term morbidity, yet structural brain injury is a rare consequence of the typical mechanics of impact in sports such as rugby, football and American football. AC is thought to increase this risk, with hypothesised mechanisms including vulnerability of blood vessels within the cyst wall, and decreased compliance of the cyst cavity in comparison to parenchyma, with a brain-fluid interface with differing capacities to absorb mechanical forces which may contribute to cyst rupture [1, 8].

From the data presented in cohort studies, the overall risk of adverse events was 1.5% (over varying follow-up intervals). This value is derived only from paediatric cohorts, and no such data have been reported for an adult cohort. Only previously untreated arachnoid cysts have been included in some observational studies [59], however more recent works have included patients with previous surgical intervention for AC who have gone on to play contact sports [1, 9]. There were no reports available from the identified case studies describing the proportion of those presenting with rupture or haemorrhage that had undergone surgical intervention for their AC prior to the index event.

The sports of those with structural brain injury in the cases discussed here are broad, and include sports not typically associated with risk of concussion or minor head impact (athletics, physical training, outdoor pursuits, swimming [14, 21, 28, 33, 41]). As described in Table 1, several reported cases recalled no preceding head injury, only a significant sporting history, and hypothesise that other elements of the sporting endeavour (via strain or Valsalva) may have been the cause of rupture or haemorrhage. Notably non-sports related recreational activities have also been linked to AC complications, including rollercoaster rides [64], and the use of vibrating belts on the head resulting in chronic subdural haematoma (CSDH) from the minimal impact injury [65].

Previous case reports and series extensively report incidences of structural brain injuries in athletes associated with incidental ACs as a result of a minor head impact or concussion. In contrast, this review identified only one report of an adult athlete with known AC whom had been exposed to contact sports and/or sports-related concussions and had not sustained a structural or surgical brain injury [58]. The remaining experiences reported in the literature of exposure to contact sports with known AC is isolated to paediatric cohorts [1, 9, 59–61]. However, based upon the lower occurrence of AC complications in these observational studies, and in this case series, it is likely that there is a considerable overrepresentation of complications in the case literature, owing to the nature of case reports as a means to publish rarer phenomena.

There are no clear clinical or radiological features based on our case series or the wider literature which appear to confer a greater degree of risk of adverse events for those with AC after sports participation. In our series, the largest AC was the only case which had previously required intervention after rupture, yet across the reported case studies there were reports of rupture in cases of all Galassi grades (I–III) (though it is noteworthy that there are no case reports of complications associated with AC in the posterior fossa or anatomical locations other than the middle fossa). In paediatric (non-sporting) settings, Galassi grade/size (along with perinatal variables) appear to confer risk of spontaneous or traumatic rupture or haemorrhage [66, 67], though there is insufficient evidence in sports upon which to draw firm conclusions.

It is notable that, amongst the athletes in this cohort whom returned to play their sport, RTP times were delayed, predominantly due to persistent symptoms and failure to progress. Whilst this is a small cohort, it suggests a possibility that athletes with AC may represent a risk factor for experience of more prolonged symptoms. However, this is a tentative observation and requires much further investigation through large adult prospective cohort studies.

### Limitations

This is a small cohort of adult players and any extrapolation to wider populations should be approached with caution. Similarly, all players presented to the sports concussion clinic due to some clinical concern, and as such this cohort represents a more specific cohort than a broad snapshot of all athletes, or even all athletes whom have sustained concussion. More widely, the literature review is likely limited by publication bias. The predilection for reporting of phenomena in case reports has likely produced an evidence base where adverse events are overrepresented. Conversely, injuries associated with AC which resulted in more severe or unfavourable outcomes than those identified here may be underreported, perhaps due to difficulties obtaining family assent for publication of such case reports; or reluctance of surgical units to report adverse outcomes.

### Clinical Implications

Conveying risk of contact-sport participation to athletes with incidental uncomplicated AC is a challenging clinical scenario. Due to the relative rarity of AC, (and within this group the relative rarity of complications), estimation of risk is difficult to establish with any degree of confidence, except to say that the risk is likely to be low. It is apparent from the prevalence of the reports identified here, that the risk of SDH or hygroma is likely to be marginally above that of other players without AC (though this in itself is a very rare phenomenon). The largest AC (Galassi III) and those which have previously required intervention may confer greater risk than smaller grades, though this is not demonstrable based on the available data. In our practice, individual surgical features of each AC allow further risk individualisation based on clinical reasoning (anatomical location; scalloping of the calvarium; position of the middle cerebral artery/bridging veins; mass effect on adjacent brain; any evidence from serial imaging; and previous neurosurgical intervention). As such, risk management approaches to asymptomatic AC vary significantly between clinicians and services. Particular sporting regulatory bodies such as the British Boxing Board of Control do not permit participation where an AC (including asymptomatic incidental lesions) has been identified, although even this position differs across combat sports internationally [68]. Across sports, clinical opinions are mixed and range from permitting any ongoing participation with no adaptation; to advising retirement [5, 41, 69, 70].

Though this work has not identified any evidence to support an absolute rule for advising against sport participation in general for those with AC, cases should be reviewed and considered on their particular features by a neurosurgeon, ideally with a specialist interest in sports-related concussion. Advice given should reflect their clinical judgement and be individualised to the player based on: the patient’s personal circumstances; AC morphology/anatomy; clinical history and clinical features. This should be offered in a nuanced consultation to support players to make informed and reasoned decisions about ongoing sports participation. The findings reported here provide tentative reassurance to support the notion that complications of AC as a result of sports injury are a rare phenomenon, and where they do occur appear to result in complete neurological recovery. This applies only to sport in general, and the content of this discussion and any clinical decision-making will be significantly impacted by the athlete’s sport, particularly in boxing and other combat sports [68].

As with contact sports participation more generally, a related question to quantification of the *level of risk* is understanding what constitutes an *acceptable level of risk.* For absolute mitigation of risk, contact sports should be avoided by all, along with many seemingly benign recreational activities which are known to (albeit infrequently) cause harm. However, given the personal and physiological potential for benefit to be derived from sports participation at any level, this approach is unreasonably restrictive. As described above, innocuous physical or recreational activities (such as swimming, athletics or physical training [14, 21, 28, 33, 41]) have been reported to also result in complications of AC. As such, complete removal of risk would be unreasonably restrictive and would be detrimental to personal health and wellbeing. In contrast, balancing the personal risk and rewards of sport cannot be determined by the clinician in isolation: beyond promotion of physical activity, many of the benefits derived from sport vary significantly between players, particularly at an amateur level. Similarly the personal risk posed from requiring surgery following a potential future complication varies between players, for example the necessity to retain a driving licence for work, or the need to provide care to dependents. In the absence of rare contraindicating features, the approach advocated by the authors is to offer an accessible summary of the evidence of the potential risk to players, in order to guide a player-led decision supported by the clinician.

### Future Directions

Amongst the many reasonable measures to improve safety in the future of contact sports, it is key to improve knowledge to ensure that players are able choose whether or not to continue to participate based on the best available information on their personal level of risk, including how this may be affected by the presence of congenital or acquired brain lesions. Prospective cohort studies from multiple centres (spanning children and adults) with known AC would be required to have extensive follow-up across their playing careers, in order to formally quantify the risk, though given the rarity of both phenomena this would be a costly endeavour which would be difficult to justify. Alternatively, increased reporting of experience of players whom continue to engage in contact sports with a known AC, as in this work, may allow for balanced literature summaries to be produced in the future.

## Conclusions

Based upon our local experience, combined with cases and cohorts reported in the literature, complications of AC after sports-related head injury appear to be relatively rare. Although outcomes were inconsistently available from the wider literature, no athletes were reported to have unfavourable outcomes after any complication, as is the case for our case series reported here. The majority in our cohort had sustained serial concussions which did not result in apparent AC complication. The single complication within this cohort occurred in the largest AC, and AC size is proposed as a possible factor associated with increased risk of contact sports participation. In this case, and across the wider published literature, reported outcomes of AC-associated structural brain lesions do not appear to be associated with poor neurological recovery. Individualised discussion on the potential risks of contact sports participation should be offered to all players with known AC, and continued high-quality observational research is encouraged to improve the information upon which shared decision-making may be made in the future.

## Data Availability

Data supporting this manuscript is available on reasonable request. Due to the identifiable nature of MRI imaging, these are not suitable to be shared.

## Abbreviations

AC: Arachnoid cyst
CSDH: Chronic subdural haematoma
IH: Interhemispheric
IV: Intraventricular
MF: Middle fossa
MRI: Magnetic resonance imaging
N/A: Not applicable
NR: Not recorded
PF: Posterior fossa
RTP: Return to play
SDH: Subdural haematoma
